# *catena*-Poly[[[diaqua­copper(II)]-bis­[μ-1,5-bis­(1*H*-imidazol-1-yl)pentane-κ^2^ *N* ^3^:*N* ^3′^]] naphthalene-1,5-disulfonate]

**DOI:** 10.1107/S1600536812045679

**Published:** 2012-11-17

**Authors:** Lai-Ping Zhang, Shu-Tang Wen, Xiao-Ning Fu

**Affiliations:** a Department of Chemistry and Chemical Engineering, Xinxiang University, Xinxiang 453000, People’s Republic of China

## Abstract

In the title complex, {[Cu(C_11_H_16_N_4_)_2_(H_2_O)_2_](C_10_H_6_O_6_S_2_)}_*n*_, the Cu^II^ atom, lying on an inversion center, is six-coordinated by two water mol­ecules and four N atoms from four 1,5-bis­(1*H*-imidazol-1-yl)pentane (biim-5) ligands in a distorted octa­hedral geometry. Adjacent Cu^II^ atoms are linked by two biim-5 ligands, forming a chain along [111]. Two atoms of the pentane group are disordered over two sets of sites, with an occupancy ratio of 0.554 (18):0.446 (18). Inter­molecular O—H⋯O hydrogen bonds link the chains and the centrosymmetric naphthalene-1,5-disulfonate anions into a layer structure parallel to (0-11).

## Related literature

For background to metal-organic coordination polymers with *N*-donor ligands, see: Kesanli *et al.* (2005 ▶); Wei *et al.* (2008 ▶); Zhang *et al.* (2010 ▶).
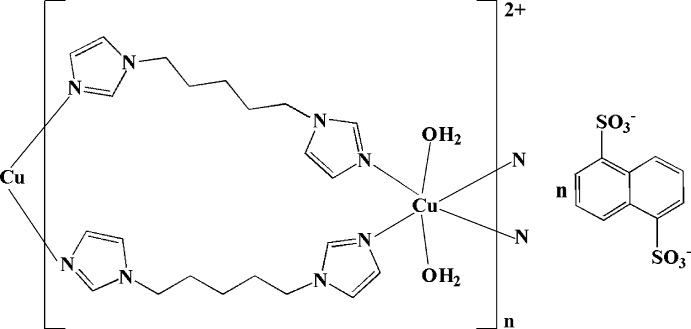


## Experimental

### 

#### Crystal data

[Cu(C_11_H_16_N_4_)_2_(H_2_O)_2_](C_10_H_6_O_6_S_2_)
*M* *_r_* = 790.37
Triclinic, 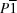

*a* = 9.300 (5) Å
*b* = 9.880 (5) Å
*c* = 11.020 (5) Å
α = 95.490 (5)°
β = 102.930 (5)°
γ = 114.000 (5)°
*V* = 881.5 (8) Å^3^
*Z* = 1
Mo *K*α radiation
μ = 0.80 mm^−1^
*T* = 293 K
0.41 × 0.33 × 0.21 mm

#### Data collection

Rigaku R-AXIS RAPID diffractometer
Absorption correction: multi-scan (*ABSCOR*; Higashi, 1995 ▶) *T*_min_ = 0.970, *T*_max_ = 0.980
8706 measured reflections
3995 independent reflections
2218 reflections with *I* > 2σ(*I*)
*R*_int_ = 0.076

#### Refinement

*R*[*F* ^2^ > 2σ(*F* ^2^)] = 0.074
*wR*(*F* ^2^) = 0.167
*S* = 1.03
3995 reflections
258 parameters
4 restraints
H atoms treated by a mixture of independent and constrained refinement
Δρ_max_ = 0.52 e Å^−3^
Δρ_min_ = −0.62 e Å^−3^

### 

Data collection: *RAPID-AUTO* (Rigaku, 1998 ▶); cell refinement: *RAPID-AUTO*; data reduction: *CrystalStructure* (Rigaku/MSC, 2002 ▶); program(s) used to solve structure: *SHELXS97* (Sheldrick, 2008 ▶); program(s) used to refine structure: *SHELXL97* (Sheldrick, 2008 ▶); molecular graphics: *XP* in *SHELXTL* (Sheldrick, 2008 ▶); software used to prepare material for publication: *SHELXTL*.

## Supplementary Material

Crystal structure: contains datablock(s) I, global. DOI: 10.1107/S1600536812045679/hy2599sup1.cif

Structure factors: contains datablock(s) I. DOI: 10.1107/S1600536812045679/hy2599Isup2.hkl

Additional supplementary materials:  crystallographic information; 3D view; checkCIF report

## Figures and Tables

**Table 1:** Hydrogen-bond geometry (Å, °)

*D*—H⋯*A*	*D*—H	H⋯*A*	*D*⋯*A*	*D*—H⋯*A*
O1*W*—H1*A*⋯O2	0.89 (6)	1.97 (6)	2.836 (6)	163 (6)
O1*W*—H1*B*⋯O1^i^	0.89 (4)	2.11 (4)	3.001 (6)	178 (7)

Symmetry code: (i) 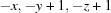.

**Fig. 1.**
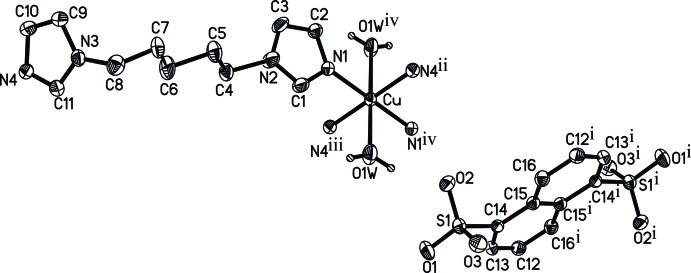
The asymmetric unit of the title compound, with displacement ellipsoids drawn at the 30% probability level. H atoms and minor disordered sites have been omitted for clarity. [Symmetry codes: (i) 1-x, 2-y, 2-z; (ii) 1+x, 1+y, 1+z; (iii) -x, -y, -z; (iv) 1-x, 1-y, 1-z.]

## supplementary crystallographic information

### Comment

There is currently much interest in adopting N-donor ligands as second ligands to prepare new metal-organic coordination polymers because of their special coordination character (Kesanli *et al.*, 2005). Among the N-donor bridging ligands, bis(imidazole) ligands, as an important family of flexible N-donor ligands, have attracted great interest. The main reason is that the flexible nature of the alkyl spacer allows the backbone of the bis(imidazole) ligand to bend and rotate freely so as to conform to the coordination geometries of central metal atoms (Wei *et al.*, 2008). As a result, the bis(imidazole) ligands, especially 1,1'-(1,4-butanediyl)bis(imidazole) (biim-4), have widely introduced into the construction of coordination polymers (Zhang *et al.*, 2010). Compared with the biim-4 ligand, 1,1'-(1,5-pentanediyl)bis(imidazole) (biim-5) ligand, bearing a longer methylene (-CH_2_-)_5_ skeleton, tends to exhibit more flexible conformations. Although compounds based on carboxylate ions and biim-5 have been reported widely, the compounds consist of sulfonate ions are relatively rare.

The asymmetric unit of the title compound contains a half of Cu^II^ ion, a half of naphthalene-1,5-disulfonate (1,5-nds) anion, one biim-5 ligand and one water molecule. As illustrated in Fig. 1, the Cu^II^ ion is six-coordinated by four N atoms from four biim-5 ligands and two water O atoms, furnishing a distorted octahedral geometry. The adjacent Cu^II^ atoms are linked by two biim-5 ligands, forming a chain along [1 1 1]. Intermolecular O—H···O hydrogen bonds link the chains and the 1,5-nds anions into a layer structure parallel to (0 -1 1).

### Experimental

A mixture of Cu(CH_3_COO)_2_.H_2_O (39.9 mg, 0.2 mmol), naphthalene-1,5-disulfonic acid (57.7 mg, 0.2 mmol) and biim-5 (41.2 mg, 0.2 mmol) was added to water (7 ml). After stirring for 15 min, the precipitate was dissolved by dropwise addition of an aqueous solution of NH_3_ (14*M*, 3 ml). Blue crystals were obtained after allowing the solution to stand at room temperature for several days.

### Refinement

All H atoms on C atoms were generated geometrically and refined as riding atoms, with C—H = 0.93 (CH) and 0.97 (CH_2_) Å and *U*_iso_(H) = 1.2*U*_eq_(C). The disorder of C4 and C5 each over two sites was refined to an occupancy ratio of 0.554 (18):0.446 (18). H atoms of water molecules were located in a difference Fourier map and refined with *U*_iso_(H) = 1.5*U*_eq_(O).

### Crystal data

**Table:**

[Cu(C_11_H_16_N_4_)_2_(H_2_O)_2_](C_10_H_6_O_6_S_2_)	*Z* = 1
*M**_r_* = 790.37	*F*(000) = 411
Triclinic, *P*1	*D*_x_ = 1.489 Mg m^−^^3^
Hall symbol: -P 1	Melting point: not measured K
*a* = 9.300 (5) Å	Mo *K*α radiation, λ = 0.71073 Å
*b* = 9.880 (5) Å	Cell parameters from 3995 reflections
*c* = 11.020 (5) Å	θ = 3.0–27.5°
α = 95.490 (5)°	µ = 0.80 mm^−^^1^
β = 102.930 (5)°	*T* = 293 K
γ = 114.000 (5)°	Block, blue
*V* = 881.5 (8) Å^3^	0.41 × 0.33 × 0.21 mm

### Data collection

**Table:**

Rigaku R-AXIS RAPID diffractometer	3995 independent reflections
Radiation source: fine-focus sealed tube	2218 reflections with *I* > 2σ(*I*)
Graphite monochromator	*R*_int_ = 0.076
Detector resolution: 10 pixels mm^-1^	θ_max_ = 27.5°, θ_min_ = 3.1°
ω scans	*h* = −12→12
Absorption correction: multi-scan (*ABSCOR*; Higashi, 1995)	*k* = −12→12
*T*_min_ = 0.970, *T*_max_ = 0.980	*l* = −13→14
8706 measured reflections	

### Refinement

**Table:**

Refinement on *F*^2^	Secondary atom site location: difference Fourier map
Least-squares matrix: full	Hydrogen site location: inferred from neighbouring sites
*R*[*F*^2^ > 2σ(*F*^2^)] = 0.074	H atoms treated by a mixture of independent and constrained refinement
*wR*(*F*^2^) = 0.167	*w* = 1/[σ^2^(*F*_o_^2^) + (0.0505*P*)^2^ + 1.4149*P*] where *P* = (*F*_o_^2^ + 2*F*_c_^2^)/3
*S* = 1.03	(Δ/σ)_max_ < 0.001
3995 reflections	Δρ_max_ = 0.52 e Å^−^^3^
258 parameters	Δρ_min_ = −0.62 e Å^−^^3^
4 restraints	Extinction correction: *SHELXL97* (Sheldrick, 2008), Fc^*^=kFc[1+0.001xFc^2^λ^3^/sin(2θ)]^-1/4^
Primary atom site location: structure-invariant direct methods	Extinction coefficient: 0.015 (3)

### Special details

**Table:**

Geometry. All e.s.d.'s (except the e.s.d. in the dihedral angle between two l.s. planes) are estimated using the full covariance matrix. The cell e.s.d.'s are taken into account individually in the estimation of e.s.d.'s in distances, angles and torsion angles; correlations between e.s.d.'s in cell parameters are only used when they are defined by crystal symmetry. An approximate (isotropic) treatment of cell e.s.d.'s is used for estimating e.s.d.'s involving l.s. planes.
Refinement. Refinement of *F*^2^ against ALL reflections. The weighted *R*-factor *wR* and goodness of fit *S* are based on *F*^2^, conventional *R*-factors *R* are based on *F*, with *F* set to zero for negative *F*^2^. The threshold expression of *F*^2^ > σ(*F*^2^) is used only for calculating *R*-factors(gt) *etc*. and is not relevant to the choice of reflections for refinement. *R*-factors based on *F*^2^ are statistically about twice as large as those based on *F*, and *R*- factors based on ALL data will be even larger.

### Fractional atomic coordinates and isotropic or equivalent isotropic displacement parameters (Å^2^)

**Table:**

	*x*	*y*	*z*	*U*_iso_*/*U*_eq_	Occ. (<1)
Cu	0.5000	0.5000	0.5000	0.0421 (3)	
C1	0.2899 (6)	0.3794 (7)	0.2360 (5)	0.0641 (17)	
H1	0.1991	0.3656	0.2646	0.077*	
C2	0.5349 (6)	0.4438 (6)	0.2291 (5)	0.0485 (13)	
H2	0.6490	0.4841	0.2538	0.058*	
C3	0.4368 (7)	0.3741 (8)	0.1108 (5)	0.0675 (18)	
H3	0.4693	0.3573	0.0391	0.081*	
C4	0.1318 (13)	0.1980 (15)	0.0189 (10)	0.053 (4)	0.554 (18)
H4A	0.1603	0.1171	−0.0048	0.063*	0.554 (18)
H4B	0.0399	0.1584	0.0543	0.063*	0.554 (18)
C5	0.0905 (13)	0.2632 (15)	−0.0932 (11)	0.058 (4)	0.554 (18)
H5A	0.1793	0.2974	−0.1322	0.069*	0.554 (18)
H5B	0.0664	0.3470	−0.0694	0.069*	0.554 (18)
C4'	0.1284 (16)	0.305 (2)	0.0057 (13)	0.056 (5)	0.446 (18)
H4'1	0.0332	0.2855	0.0362	0.068*	0.446 (18)
H4'2	0.1494	0.3908	−0.0355	0.068*	0.446 (18)
C5'	0.1049 (18)	0.1697 (19)	−0.0814 (16)	0.065 (5)	0.446 (18)
H5'1	0.1007	0.0890	−0.0365	0.078*	0.446 (18)
H5'2	0.1929	0.1935	−0.1212	0.078*	0.446 (18)
C6	−0.0690 (7)	0.1223 (9)	−0.1858 (6)	0.082 (2)	
C7	−0.1086 (8)	0.2045 (7)	−0.2907 (6)	0.078 (2)	
H7A	−0.1019	0.3000	−0.2517	0.094*	
H7B	−0.0273	0.2267	−0.3370	0.094*	
C8	−0.2770 (7)	0.1114 (6)	−0.3822 (5)	0.0565 (15)	
H8A	−0.3585	0.0944	−0.3364	0.068*	
H8B	−0.2967	0.1679	−0.4461	0.068*	
C9	−0.2189 (6)	−0.0582 (6)	−0.5301 (5)	0.0482 (13)	
H9	−0.1436	0.0150	−0.5605	0.058*	
C11	−0.3937 (6)	−0.1703 (6)	−0.4280 (5)	0.0445 (12)	
H11	−0.4599	−0.1853	−0.3738	0.053*	
C10	−0.2722 (6)	−0.2088 (6)	−0.5604 (5)	0.0511 (13)	
H10	−0.2386	−0.2577	−0.6164	0.061*	
C12	0.4074 (6)	0.7806 (6)	1.1100 (5)	0.0474 (13)	
H12	0.3943	0.7151	1.1664	0.057*	
C13	0.2935 (6)	0.7343 (5)	0.9885 (5)	0.0432 (12)	
H13	0.2051	0.6385	0.9657	0.052*	
C14	0.3102 (5)	0.8269 (5)	0.9041 (4)	0.0349 (11)	
C15	0.4423 (5)	0.9768 (5)	0.9385 (4)	0.0366 (11)	
C16	0.4633 (6)	1.0791 (6)	0.8548 (4)	0.0422 (12)	
H16	0.3876	1.0500	0.7748	0.051*	
N2	0.2814 (6)	0.3331 (7)	0.1160 (4)	0.0786 (18)	
N1	0.4413 (5)	0.4466 (4)	0.3081 (4)	0.0413 (10)	
N3	−0.2978 (5)	−0.0341 (4)	−0.4455 (4)	0.0411 (10)	
N4	−0.3829 (5)	−0.2802 (5)	−0.4970 (4)	0.0426 (10)	
O1	0.0560 (4)	0.6024 (4)	0.7469 (4)	0.0597 (10)	
O2	0.2596 (4)	0.7717 (4)	0.6604 (3)	0.0529 (10)	
O3	0.0816 (4)	0.8556 (4)	0.7419 (3)	0.0544 (10)	
S1	0.16398 (15)	0.75847 (15)	0.75061 (12)	0.0442 (4)	
O1W	0.2295 (5)	0.5212 (5)	0.4900 (4)	0.0657 (12)	
H1A	0.217 (7)	0.588 (6)	0.542 (5)	0.099*	
H1B	0.144 (5)	0.486 (7)	0.420 (3)	0.099*	

### Atomic displacement parameters (Å^2^)

**Table:**

	*U*^11^	*U*^22^	*U*^33^	*U*^12^	*U*^13^	*U*^23^
Cu	0.0430 (5)	0.0387 (5)	0.0300 (5)	0.0065 (4)	0.0085 (4)	−0.0002 (3)
C1	0.035 (3)	0.098 (5)	0.038 (3)	0.015 (3)	0.009 (3)	−0.007 (3)
C2	0.036 (3)	0.062 (4)	0.037 (3)	0.016 (2)	0.007 (2)	0.000 (2)
C3	0.055 (3)	0.102 (5)	0.032 (3)	0.025 (3)	0.015 (3)	−0.006 (3)
C4	0.054 (6)	0.051 (8)	0.035 (7)	0.012 (5)	0.002 (5)	0.004 (5)
C5	0.057 (7)	0.049 (8)	0.047 (8)	0.017 (6)	−0.007 (6)	0.004 (5)
C4'	0.046 (7)	0.068 (12)	0.039 (9)	0.023 (7)	−0.006 (6)	−0.007 (7)
C5'	0.074 (10)	0.046 (9)	0.061 (11)	0.034 (8)	−0.011 (8)	−0.009 (7)
C6	0.054 (4)	0.104 (6)	0.046 (4)	0.018 (4)	−0.020 (3)	−0.015 (3)
C7	0.070 (4)	0.050 (4)	0.074 (4)	−0.004 (3)	0.019 (4)	−0.027 (3)
C8	0.072 (4)	0.045 (3)	0.053 (3)	0.027 (3)	0.016 (3)	0.010 (3)
C9	0.049 (3)	0.046 (3)	0.045 (3)	0.012 (2)	0.021 (3)	0.009 (2)
C11	0.041 (3)	0.044 (3)	0.040 (3)	0.012 (2)	0.009 (2)	0.005 (2)
C10	0.054 (3)	0.050 (3)	0.048 (3)	0.017 (3)	0.027 (3)	0.005 (2)
C12	0.057 (3)	0.042 (3)	0.044 (3)	0.018 (3)	0.022 (3)	0.013 (2)
C13	0.042 (3)	0.032 (3)	0.048 (3)	0.010 (2)	0.016 (2)	0.002 (2)
C14	0.027 (2)	0.038 (3)	0.035 (2)	0.012 (2)	0.008 (2)	−0.001 (2)
C15	0.033 (2)	0.038 (3)	0.037 (2)	0.016 (2)	0.0092 (19)	0.003 (2)
C16	0.045 (3)	0.043 (3)	0.036 (3)	0.019 (2)	0.008 (2)	0.007 (2)
N2	0.038 (2)	0.133 (5)	0.030 (2)	0.016 (3)	0.001 (2)	−0.015 (3)
N1	0.038 (2)	0.042 (2)	0.036 (2)	0.0116 (18)	0.0102 (19)	0.0011 (17)
N3	0.040 (2)	0.038 (2)	0.035 (2)	0.0113 (19)	0.0035 (18)	0.0040 (17)
N4	0.046 (2)	0.040 (2)	0.036 (2)	0.0132 (19)	0.0129 (19)	0.0054 (18)
O1	0.048 (2)	0.045 (2)	0.059 (2)	0.0027 (17)	0.0022 (18)	0.0011 (17)
O2	0.052 (2)	0.059 (2)	0.042 (2)	0.0200 (18)	0.0161 (17)	−0.0015 (16)
O3	0.044 (2)	0.062 (2)	0.057 (2)	0.0268 (19)	0.0090 (18)	0.0094 (18)
S1	0.0364 (7)	0.0429 (8)	0.0408 (7)	0.0108 (6)	0.0052 (6)	−0.0013 (5)
O1W	0.055 (2)	0.068 (3)	0.059 (3)	0.028 (2)	−0.001 (2)	−0.015 (2)

### Geometric parameters (Å, º)

**Table:**

Cu—N4^i^	1.988 (4)	C8—N3	1.453 (6)
Cu—N1	2.021 (4)	C8—H8A	0.9700
Cu—O1W	2.587 (5)	C8—H8B	0.9700
C1—N1	1.302 (6)	C9—C10	1.343 (7)
C1—N2	1.331 (7)	C9—N3	1.367 (6)
C1—H1	0.9300	C9—H9	0.9300
C2—C3	1.339 (7)	C11—N4	1.316 (6)
C2—N1	1.368 (6)	C11—N3	1.339 (6)
C2—H2	0.9300	C11—H11	0.9300
C3—N2	1.350 (7)	C10—N4	1.368 (6)
C3—H3	0.9300	C10—H10	0.9300
C4—C5	1.49 (2)	C12—C16^ii^	1.360 (7)
C4—N2	1.553 (11)	C12—C13	1.406 (7)
C4—H4A	0.9700	C12—H12	0.9300
C4—H4B	0.9700	C13—C14	1.355 (6)
C5—C6	1.597 (12)	C13—H13	0.9300
C5—H5A	0.9700	C14—C15	1.432 (6)
C5—H5B	0.9700	C14—S1	1.781 (4)
C4'—C5'	1.48 (3)	C15—C16	1.417 (6)
C4'—N2	1.555 (14)	C15—C15^ii^	1.425 (9)
C4'—H4'1	0.9700	C16—C12^ii^	1.360 (7)
C4'—H4'2	0.9700	C16—H16	0.9300
C5'—C6	1.615 (15)	N4—Cu^iii^	1.988 (4)
C5'—H5'1	0.9700	O1—S1	1.448 (4)
C5'—H5'2	0.9700	O2—S1	1.457 (4)
C6—C7	1.537 (10)	O3—S1	1.449 (4)
C7—C8	1.504 (7)	O1W—H1A	0.89 (6)
C7—H7A	0.9700	O1W—H1B	0.89 (4)
C7—H7B	0.9700		
			
N4^i^—Cu—N4^iv^	180.000 (1)	N3—C8—C7	112.9 (5)
N4^i^—Cu—N1^v^	91.92 (16)	N3—C8—H8A	109.0
N4^iv^—Cu—N1^v^	88.08 (16)	C7—C8—H8A	109.0
N4^i^—Cu—N1	88.08 (16)	N3—C8—H8B	109.0
N4^iv^—Cu—N1	91.92 (16)	C7—C8—H8B	109.0
N1^v^—Cu—N1	180.000 (1)	H8A—C8—H8B	107.8
O1W—Cu—N1	91.80 (17)	C10—C9—N3	106.1 (5)
O1W—Cu—N4^iv^	91.02 (18)	C10—C9—H9	127.0
O1W—Cu—N4^i^	88.98 (18)	N3—C9—H9	127.0
O1W—Cu—O1W^v^	180.00	N4—C11—N3	111.7 (5)
O1W—Cu—N1^v^	88.20 (17)	N4—C11—H11	124.2
N1—C1—N2	111.5 (5)	N3—C11—H11	124.2
N1—C1—H1	124.3	C9—C10—N4	110.3 (5)
N2—C1—H1	124.3	C9—C10—H10	124.8
C3—C2—N1	109.5 (5)	N4—C10—H10	124.8
C3—C2—H2	125.3	C16^ii^—C12—C13	120.1 (5)
N1—C2—H2	125.3	C16^ii^—C12—H12	120.0
C2—C3—N2	106.4 (5)	C13—C12—H12	120.0
C2—C3—H3	126.8	C14—C13—C12	121.2 (4)
N2—C3—H3	126.8	C14—C13—H13	119.4
C5—C4—N2	104.6 (10)	C12—C13—H13	119.4
C5—C4—H4A	110.8	C13—C14—C15	120.5 (4)
N2—C4—H4A	110.8	C13—C14—S1	118.6 (4)
C5—C4—H4B	110.8	C15—C14—S1	120.9 (4)
N2—C4—H4B	110.8	C16—C15—C15^ii^	119.2 (5)
H4A—C4—H4B	108.9	C16—C15—C14	122.7 (4)
C4—C5—C6	102.3 (10)	C15^ii^—C15—C14	118.1 (5)
C4—C5—H5A	111.3	C12^ii^—C16—C15	120.9 (5)
C6—C5—H5A	111.3	C12^ii^—C16—H16	119.5
C4—C5—H5B	111.3	C15—C16—H16	119.5
C6—C5—H5B	111.3	C1—N2—C3	107.3 (4)
H5A—C5—H5B	109.2	C1—N2—C4	125.1 (6)
C5'—C4'—N2	102.7 (13)	C3—N2—C4	122.1 (6)
C5'—C4'—H4'1	111.2	C1—N2—C4'	120.2 (7)
N2—C4'—H4'1	111.2	C3—N2—C4'	127.6 (7)
C5'—C4'—H4'2	111.2	C1—N1—C2	105.4 (4)
N2—C4'—H4'2	111.2	C1—N1—Cu	122.5 (4)
H4'1—C4'—H4'2	109.1	C2—N1—Cu	131.1 (3)
C4'—C5'—C6	103.8 (12)	C11—N3—C9	106.9 (4)
C4'—C5'—H5'1	111.0	C11—N3—C8	126.2 (5)
C6—C5'—H5'1	111.0	C9—N3—C8	126.8 (5)
C4'—C5'—H5'2	111.0	C11—N4—C10	105.0 (4)
C6—C5'—H5'2	111.0	C11—N4—Cu^iii^	126.0 (4)
H5'1—C5'—H5'2	109.0	C10—N4—Cu^iii^	129.0 (4)
C7—C6—C5	97.8 (7)	O1—S1—O3	113.4 (2)
C7—C6—C5'	128.2 (9)	O1—S1—O2	112.4 (2)
C8—C7—C6	112.0 (5)	O3—S1—O2	113.0 (2)
C8—C7—H7A	109.2	O1—S1—C14	105.8 (2)
C6—C7—H7A	109.2	O3—S1—C14	105.9 (2)
C8—C7—H7B	109.2	O2—S1—C14	105.5 (2)
C6—C7—H7B	109.2	H1A—O1W—H1B	107 (5)
H7A—C7—H7B	107.9		
			
N1—C2—C3—N2	−0.1 (7)	C5—C4—N2—C4'	28.3 (11)
N2—C4—C5—C6	−177.0 (7)	C5'—C4'—N2—C1	−142.0 (11)
N2—C4'—C5'—C6	171.5 (9)	C5'—C4'—N2—C3	66.1 (17)
C4—C5—C6—C7	175.4 (10)	C5'—C4'—N2—C4	−31.9 (11)
C4—C5—C6—C5'	−34.7 (13)	N2—C1—N1—C2	0.5 (7)
C4'—C5'—C6—C7	70.9 (16)	N2—C1—N1—Cu	−169.3 (4)
C4'—C5'—C6—C5	31.7 (12)	C3—C2—N1—C1	−0.2 (7)
C5—C6—C7—C8	−167.3 (7)	C3—C2—N1—Cu	168.3 (4)
C5'—C6—C7—C8	170.4 (9)	N4^i^—Cu—N1—C1	61.3 (5)
C6—C7—C8—N3	−59.1 (7)	N4^iv^—Cu—N1—C1	−118.7 (5)
N3—C9—C10—N4	0.1 (6)	N4^i^—Cu—N1—C2	−105.5 (5)
C16^ii^—C12—C13—C14	0.5 (8)	N4^iv^—Cu—N1—C2	74.5 (5)
C12—C13—C14—C15	−2.0 (7)	N4—C11—N3—C9	−0.7 (5)
C12—C13—C14—S1	178.4 (4)	N4—C11—N3—C8	−178.5 (4)
C13—C14—C15—C16	−178.6 (5)	C10—C9—N3—C11	0.3 (5)
S1—C14—C15—C16	0.9 (6)	C10—C9—N3—C8	178.1 (5)
C13—C14—C15—C15^ii^	2.1 (8)	C7—C8—N3—C11	111.1 (6)
S1—C14—C15—C15^ii^	−178.3 (4)	C7—C8—N3—C9	−66.2 (7)
C15^ii^—C15—C16—C12^ii^	0.8 (8)	N3—C11—N4—C10	0.7 (5)
C14—C15—C16—C12^ii^	−178.5 (5)	N3—C11—N4—Cu^iii^	179.0 (3)
N1—C1—N2—C3	−0.5 (8)	C9—C10—N4—C11	−0.5 (6)
N1—C1—N2—C4	153.4 (8)	C9—C10—N4—Cu^iii^	−178.7 (3)
N1—C1—N2—C4'	−157.5 (9)	C13—C14—S1—O1	−3.7 (5)
C2—C3—N2—C1	0.3 (8)	C15—C14—S1—O1	176.8 (4)
C2—C3—N2—C4	−154.5 (8)	C13—C14—S1—O3	117.0 (4)
C2—C3—N2—C4'	155.1 (10)	C15—C14—S1—O3	−62.6 (4)
C5—C4—N2—C1	125.9 (9)	C13—C14—S1—O2	−123.0 (4)
C5—C4—N2—C3	−83.8 (12)	C15—C14—S1—O2	57.4 (4)

Symmetry codes: (i) −*x*, −*y*, −*z*; (ii) −*x*+1, −*y*+2, −*z*+2; (iii) *x*−1, *y*−1, *z*−1; (iv) *x*+1, *y*+1, *z*+1; (v) −*x*+1, −*y*+1, −*z*+1.

### Hydrogen-bond geometry (Å, º)

**Table:**

*D*—H···*A*	*D*—H	H···*A*	*D*···*A*	*D*—H···*A*
O1*W*—H1*A*···O2	0.89 (6)	1.97 (6)	2.836 (6)	163 (6)
O1*W*—H1*B*···O1^vi^	0.89 (4)	2.11 (4)	3.001 (6)	178 (7)

Symmetry code: (vi) −*x*, −*y*+1, −*z*+1.
